# Branched-Chain Amino Acid Deprivation Decreases Lipid Oxidation and Lipogenesis in C2C12 Myotubes

**DOI:** 10.3390/metabo12040328

**Published:** 2022-04-05

**Authors:** Sira Karvinen, Vasco Fachada, Ulla-Maria Sahinaho, Satu Pekkala, Juulia H. Lautaoja, Sakari Mäntyselkä, Perttu Permi, Juha J. Hulmi, Mika Silvennoinen, Heikki Kainulainen

**Affiliations:** 1 NeuroMuscular Research Center, Faculty of Sport and Health Sciences, University of Jyväskylä, FI-40014 Jyväskylä, Finland; sira.m.karvinen@jyu.fi (S.K.); vasco.fachada@gmail.com (V.F.); ulla-maria.u-m.sahinaho@jyu.fi (U.-M.S.); satu.p.pekkala@jyu.fi (S.P.); juulia.h.lautaoja@jyu.fi (J.H.L.); sakari.a.mantyselka@jyu.fi (S.M.); juha.hulmi@jyu.fi (J.J.H.); mmjsilvennoinen@gmail.com (M.S.); 2 Department of Biological and Environmental Science, University of Jyväskylä, FI-40014 Jyväskylä, Finland; perttu.permi@jyu.fi; 3 Department of Chemistry, Nanoscience Center, University of Jyväskylä, FI-40014 Jyväskylä, Finland; 4 Institute of Biotechnology, Helsinki Institute of Life Science, University of Helsinki, FI-00014 Helsinki, Finland

**Keywords:** metabolic health, protein supplementation, skeletal muscle, electrical pulse stimulation, in vitro exercise, nuclear magnetic resonance spectroscopy

## Abstract

Impaired lipid metabolism is a common risk factor underlying several metabolic diseases such as metabolic syndrome and type 2 diabetes. Branched-chain amino acids (BCAAs) that include valine, leucine and isoleucine have been proven to share a role in lipid metabolism and hence in maintaining metabolic health. We have previously introduced a hypothesis suggesting that BCAA degradation mechanistically connects to lipid oxidation and storage in skeletal muscle. To test our hypothesis, the present study examined the effects of BCAA deprivation and supplementation on lipid oxidation, lipogenesis and lipid droplet characteristics in murine C2C12 myotubes. In addition, the role of myotube contractions on cell metabolism was studied by utilizing in vitro skeletal-muscle-specific exercise-like electrical pulse stimulation (EPS). Our results showed that the deprivation of BCAAs decreased both lipid oxidation and lipogenesis in C2C12 myotubes. BCAA deprivation further diminished the number of lipid droplets in the EPS-treated myotubes. EPS decreased lipid oxidation especially when combined with high BCAA supplementation. Similar to BCAA deprivation, high BCAA supplementation also decreased lipid oxidation. The present results highlight the role of an adequate level of BCAAs in healthy lipid metabolism.

## 1. Introduction

Impaired lipid metabolism and fat accumulation predispose people to several adverse health risks such as insulin resistance, metabolic syndrome and type 2 diabetes [1]. Over the last decade, branched-chain amino acids (BCAAs) that include valine, leucine and isoleucine have been proven to share a role in fat oxidation and hence in maintaining metabolic health [2,3]. More specifically, metabolomic and transcriptomic studies suggest that efficient BCAA catabolism (i.e., low serum BCAA levels) is associated with higher fat oxidation, physical activity and leanness [4,5]. On the contrary, inefficient BCAA catabolism (i.e., high serum BCAA levels) is linked to low physical activity, increased adiposity and other risk factors for metabolic diseases [6]. Nevertheless, the underlying mechanisms between BCAA utilization and lipid oxidation remain to be determined.

**Figure 1:**
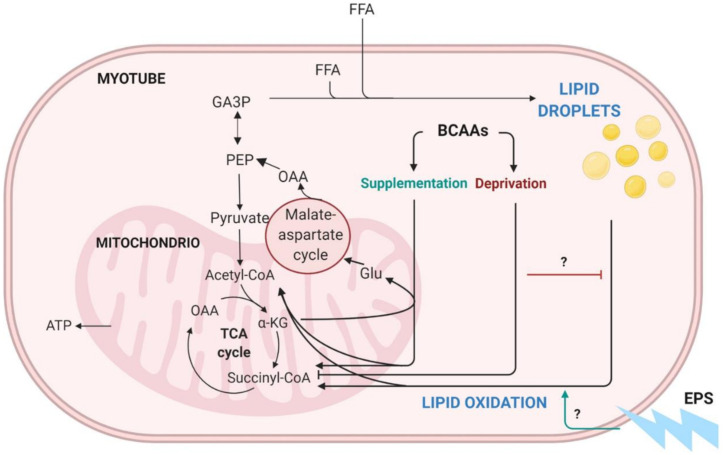
Schematic presentation of BCAA catabolism, lipid oxidation and lipogenesis in skeletal muscle. BCAA = branched-chain amino acid, EPS = electrical pulse stimulation, TCA = tricarboxylic acid cycle, OAA = oxaloacetate, CoA = coenzyme A, ATP = adenosine triphosphate, αKG = α-ketoglutarate, Glu = glucose, PEP = phosphoenolpyruvate, GAP3 = glyceraldehyde-3-phosphate, FFA = free fatty acid. Modified from Kainulainen et al., 2013 [3]. Figure was created with BioRender.com.

We have previously introduced a hypothesis suggesting that BCAA degradation mechanistically connects to lipid oxidation and storage in the skeletal muscle (Figure 1) [3]. Unlike other amino acids, BCAAs are not degraded directly by the liver but are transported into the bloodstream and catabolized mainly in muscle cells [7,8]. Since skeletal muscle comprises around 40% of body mass, it is also the main tissue that oxidizes lipids and thus aids in the prevention of obesity [9,10]. According to our hypothesis, we propose that, especially during increased demand for BCAA catabolism (such as during exercise), transamination of BCAAs is critical for cytosolic oxaloacetate formation. In our model, oxaloacetate is further metabolized to phosphoenolpyruvate for glyceroneogenesis, which is required in skeletal muscles for the storage of fatty acids as lipid droplets (LDs) (Figure 1). We suggest that these intramyocellular LDs are then utilized to provide energy during extended exercise sessions as well as constitutively during rest. Thus, the high activity of this cycle leads to better exercise performance, leaner body composition and improved health [3]. Our hypothesis is supported by the observations that the prevention of amino acid catabolism in mice impairs exercise metabolism and reduces endurance capacity [11], whereas high expression of the genes involved in BCAA degradation and fatty acid metabolism in skeletal muscle is linked to high endurance capacity in rats [12]. Furthermore, acute exercise stimulus has proven to activate branched-chain α-ketoacid dehydrogenase (BCKD) [13,14], which is the main regulator of BCAA oxidation.

In line with our hypothesis, a growing number of both rodent and human studies suggest that BCAA-rich protein supplementation has beneficial effects on several health-and-fitness-related factors, such as body composition, exercise performance and muscle properties, as well as glucose and lipid metabolism [15,16,17,18,19,20,21,22]. On the other hand, BCAA deficiency has been shown to result in impaired growth and protein wasting, as well as changes in hormonal secretion and intracellular signaling [23,24]. In contrast, some studies have shown that diets with reduced BCAA content promote metabolic health [25,26]. Particularly, reducing isoleucine has been shown to restore the metabolic health of diet-induced obese mice [27].

Exercise acutely increases fat oxidation in the skeletal muscles [28]. Furthermore, endurance training increases the capacity of muscle to oxidize fat by increasing mitochondrial density, the activity of key enzymes involved in lipid oxidation and oxygen delivery to the muscles [29]. It is also well-established that skeletal muscles from trained individuals have higher lipid content compared with untrained individuals, yet they have better insulin sensitivity and oxidative capacity [30,31]. This phenomenon, called “athlete’s paradox”, is thought to store and serve energy in the form of LDs for long-lasting exercise [30]. Some studies suggest that BCAA supplementation may further promote resistance to fatigue by increasing lipid oxidation during exercise [16].

In the present study, we examined the effects of BCAA deprivation and supplementation on lipid metabolism in murine C2C12 myotubes. In addition, we utilized an in vitro skeletal-muscle-specific exercise-like electrical pulse stimulation (EPS) to mimic and investigate the effect of exercise on myotube metabolism. According to the previous literature, we suggested two hypotheses: First, deprivation of BCAAs will lead to reduced lipid oxidation in myotubes. Second, EPS combined with BCAA supplementation further increases lipid oxidation in myotubes. In addition, we investigated how these conditions affect lipogenesis and lipid droplets (Figure 1).

## 2. Results

**Figure 2:**
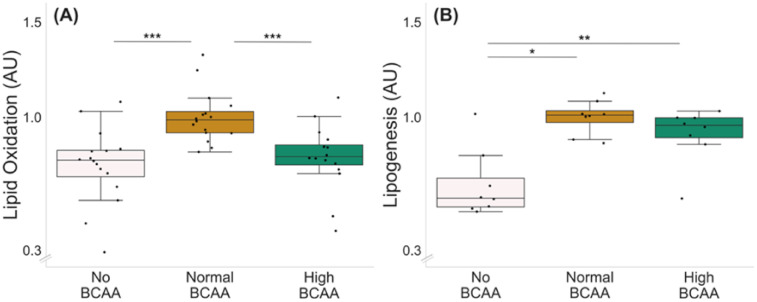
Lipid oxidation (**A**) and lipogenesis (**B**) in C2C12 myotubes with different BCAA levels. (**A**) Normal BCAA level (0.8 mmol/L) led to higher lipid oxidation compared with no BCAA (0 mmol/L) or high BCAA (2.8 mmol/L). *n* = 16/group. (**B**) Normal and high BCAA levels led to higher lipogenesis compared with no BCAA. *n* = 8/group. Boxes in the boxplot figures depict interquartile ranges and medians, and whiskers represent the 95% confidence interval. * *p* ≤ 0.050, ** *p* ≤ 0.010, *** *p* ≤ 0.001. BCAA = branched-chain amino acid.

Optimal BCAA supplementation for lipid oxidation was tested with three different BCAA concentrations: 0 (no BCAA), 0.8 (normal BCAA) and 2.8 mmol/L (high BCAA) (Figure 2A). Of the three BCAA concentrations tested, the normal BCAA led to the highest lipid oxidation compared with both no BCAA and high BCAA groups (Figure 2A, *p* ≤ 0.001); thus, it was chosen as the control level for the lipid oxidation experiments.

The optimal BCAA supplementation for lipogenesis was examined with the same BCAA concentrations as used for the lipid oxidation experiment (Figure 2B). Of the BCAA concentrations tested, both normal and high BCAA led to higher lipogenesis compared with no BCAA (Figure 2B, *p* ≤ 0.021). Since the groups with normal and high supplementation of BCAAs did not significantly differ from each other (*p* = 0.093), the same concentration as chosen for the oxidation experiments was used as the control level for the lipogenesis experiments.

### 2.1. BCAA Deprivation Decreased Lipid Oxidation and Lipogenesis in C2C12 Myotubes

**Figure 3:**
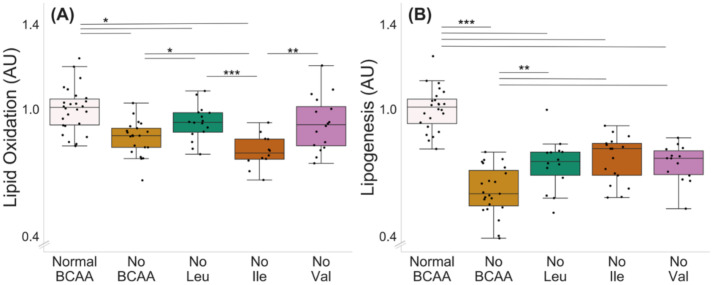
Lipid oxidation (**A**) and lipogenesis (**B**) with BCAA deprivation in murine C2C12 myotubes. (**A**) Deprivation of all BCAAs or deprivation of leucine or isoleucine alone reduced lipid oxidation in myotubes. Lipid oxidation was lower in isoleucine deprivation group compared with leucine and valine deprivation groups. Normal BCAA *n* = 26, no BCAA *n* = 21, no Leu *n* = 16, no Ile *n* = 12, no Val *n* = 16. (**B**) Deprivation of all BCAAs reduced lipogenesis in myotubes (normal BCAA vs. no BCAA). Deprivation of a single BCAA reduced lipogenesis compared with normal BCAA. Lipogenesis in single BCAA deprivation groups was higher compared with the no BCAA group. Normal BCAA *n* = 24, no BCAA *n* = 23, no Leu *n* = 14, no Ile *n* = 17, no Val *n* = 14. Boxes in the boxplot figures depict interquartile ranges and medians, and whiskers represent the 95% confidence interval. * *p* ≤ 0.050, ** *p* ≤ 0.010, *** *p* ≤ 0.001. BCAA = branched-chain amino acid, Leu = leucine, Ile = isoleucine, Val = valine.

Deprivation of all BCAAs decreased lipid oxidation in murine C2C12 myotubes (normal BCAA vs. no BCAA, *p* < 0.001, Figure 3A). In addition, the deprivation of leucine (*p* = 0.046) and isoleucine (*p* < 0.001), but not valine (0 = 0.070), decreased lipid oxidation. Lipid oxidation was also lower in the isoleucine deprivation group compared with the leucine and valine deprivation groups (*p* ≤ 0.004, Figure 3A). The deprivation of all BCAAs decreased lipogenesis in the myotubes (Figure 3B, normal BCAA vs. no BCAA, *p* < 0.001). In addition, the deprivation of a single BCAA at a time (valine, leucine or isoleucine) decreased lipogenesis compared with normal BCAA (*p* < 0.001), yet the decrease was not as significant as when all BCAAs were absent (no Leu, no Ile or no Val compared with no BCAA, *p* ≤ 0.003).

### 2.2. High BCAA Supplementation Combined with EPS Decreased Lipid Oxidation, whereas BCAA Deprivation but Not EPS Decreased Lipogenesis in C2C12 Myotubes

**Figure 4:**
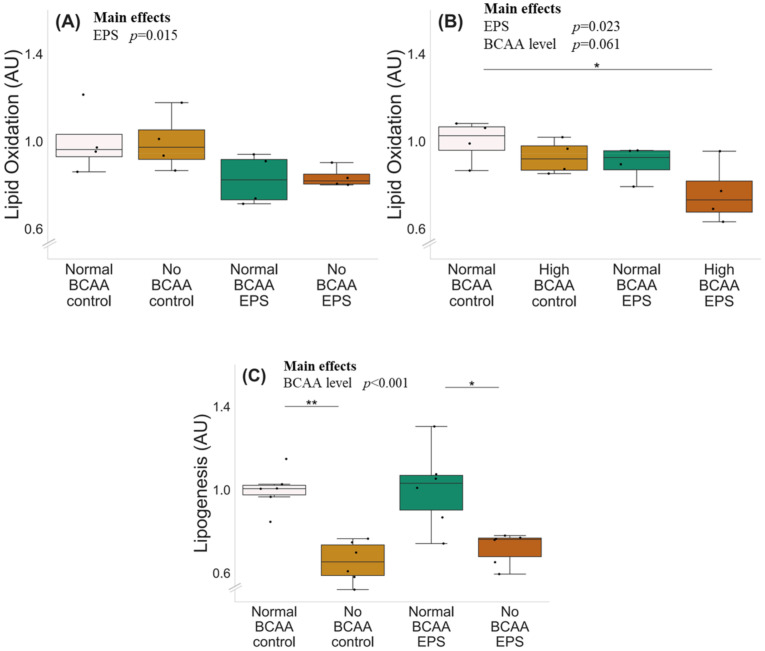
Lipid oxidation, BCAA deprivation and EPS in murine C2C12 myotubes with normal (**A**) and high (**B**) BCAA levels and lipogenesis with BCAA deprivation and EPS in myotubes (**C**). (**A**) Normal BCAA (0.8 mmol/L) concentration, EPS or BCAA deprivation did not affect lipid oxidation. (**B**) High BCAA (2.8 mmol/L) combined with EPS-reduced lipid oxidation. *n* = 4/group. (**C**) Deprivation of BCAAs reduced lipogenesis in myotubes during control conditions (normal BCAA vs. no BCAA) and with EPS treatment (normal BCAA EPS vs. no BCAA EPS) *n* = 6/group. Boxes in the boxplot figures depict interquartile ranges and medians, and whiskers represent the 95% confidence interval, * *p* ≤ 0.050, ** *p* ≤ 0.010. BCAA = branched-chain amino acid, EPS = skeletal-muscle-specific exercise-like electrical pulse stimulation.

The experiments with normal (0.8 mmol/L), no (0 mmol/L) and high (2.8 mmol/L) BCAA supplementation with and without EPS are shown in Figure 4. There was a significant effect of EPS regardless of BCAA level (*p* ≤ 0.023), indicating that EPS decreased lipid oxidation (Figure 4A,B). High BCAA content in cell media did not affect lipid oxidation (normal BCAA control vs. high BCAA control, *p* = 0.292), whereas high BCAA combined with EPS decreased lipid oxidation (*p* = 0.032). The deprivation of BCAAs decreased lipogenesis in both control (*p* = 0.002) and EPS-treated (*p* = 0.022) myotubes (Figure 4C).

### 2.3. BCAA Deprivation Diminished the Number of Lipid Droplets in the EPS-Treated C2C12 Myotubes

**Figure 5:**
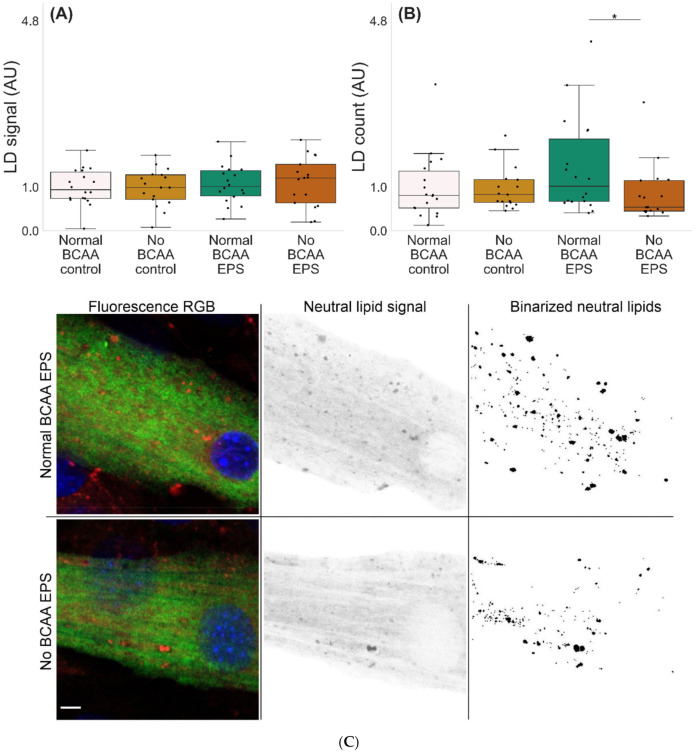
Lipid droplet characteristics in murine C2C12 myotubes (**A**–**C**). (**A**) In EPS-treated myotubes, there were no changes in LD signal fraction, whereas (**B**,**C**) BCAA deprivation diminished the number of LDs (EPS-treated myotubes 0.8 mmol BCAA vs. 0 mmol/L BCAA). *n* = 17–18/group. Boxes in the boxplot figures depict interquartile ranges and medians, and whiskers represent the 95% confidence interval, * *p* ≤ 0.050. BCAA = branched-chain amino acid, EPS = skeletal-muscle-specific exercise-like electrical pulse stimulation. In (**C**), MF20 = green, DAPI = blue and LD540 = red, gray and binary. Scale bar = 5 µm.

Microscopic examinations of lipid droplets (LD) in murine C2C12 myotubes with and without BCAA and with and without EPS are shown in Figure 5. There were no significant effects of EPS, BCAA levels or their interaction. However, the group-wise comparisons showed that in EPS-treated myotubes, BCAA deprivation led to a lower number of LDs per mm^3^ (*p* = 0.048, Figure 5B,C).

### 2.4. Metabolites in C2C12 Myotubes and in Cell Culture Media

To confirm that our cell culture media with no BCAA were very low of BCAAs and that being supplemented with normal BCAA had substantially higher BCAA and BCAA degradation product contents, nuclear magnetic resonance (NMR)-based metabolomics was conducted for murine C2C12 myotubes and cell media (Supplementary Table S1). Our results confirmed that, in myotubes cultured without BCAAs (no BCAA), the levels of pooled BCAAs (isoleucine, leucine and valine) and individual BCAAs were lower than in myotubes cultured with BCAAs (normal BCAA) (*p* = 0.004, Supplementary Table S1). Furthermore, in the cell culture media of the myotubes cultured without BCAAs, the level of pooled BCAAs, individual BCAAs as well as pooled BCAA degradation products (isobutyrate, isovalerate, 2-ketoisovalerate, 2-methylbutyrate, 3-methyl-2-oxovalerate and 2-oxoisocaproate) were lower than in myotubes cultured with BCAAs, suggesting decreased BCAA degradation in cells treated with no BCAA media (*p* = 0.014, Supplementary Table S1). All identified metabolites in myotubes and cell culture media are presented in Supplementary Table S2.

### 2.5. Total Protein Content, and Citrate Synthase Activity and Cell Viability Measurements

Corresponding total protein contents and citrate synthase activities of the study setups are presented in Supplementary Figures S1–S3. Overall, BCAA supplementation increased both the total protein content and CS activity of the murine C2C12 myotubes (Supplementary Figures S1–S3).

The cell viability following EPS was examined via measuring lactate dehydrogenase (LDH) level in cell medium after lipid oxidation and lipogenesis experiments (Supplementary Figure S4). Our results showed that, in lipid oxidation experiment, the LDH level was lower in the media of EPS-treated myotubes (main effect *p* = 0.013), whereas there was no effect of BCAA level (main effect *p* = 0.257, Supplementary Figure S4a). When examining the lipogenesis experiment, BCAA deprivation increased the LDH level (main effect *p* = 0.003). EPS treatment slightly but consistently increased LDH activity in all of the studied BCAA concentrations in the lipogenesis experiment (main effect *p* > 0.001).

## 3. Discussion

This study examined the effects of branched-chain amino acid (BCAAs = valine, leucine and isoleucine) supplementation on lipid metabolism of the murine C2C12 myotubes. In addition, the effects of muscle contractions in muscle metabolism were mimicked and studied in myotubes utilizing in vitro skeletal-muscle-specific exercise-like electrical pulse stimulation (EPS). Supporting our previous hypothesis [3], the present study showed that deprivation of the BCAAs reduced both lipid oxidation and lipogenesis in C2C12 myotubes. The BCAA deprivation further diminished the number of lipid droplets in the EPS-treated myotubes. High serum BCAA levels can act as biomarkers for some metabolic diseases [4,6]; indeed, we observed that a high BCAA level in myotube media may lead to dysregulated lipid metabolism. However, the current results together with the data from other research groups [32] suggest that also very low levels (or ingestion of) BCAAs may lead to disturbances in muscle lipid metabolism. These results thus suggest that adequate (not too high nor too low) BCAA level is important for healthy lipid metabolism in muscle. However, further studies are warranted to elucidate the dose response in BCAA ingestion and its possible connection to lipid metabolisms in vivo.

### 3.1. The Deprivation of BCAAs Reduced Lipid Oxidation and Lipogenesis in C2C12 Myotubes

We hypothesized that as BCAAs may feed lipid oxidation, deprivation of BCAAs would lead to reduced lipid oxidation. According to our hypothesis, deprivation of all BCAAs reduced lipid oxidation in murine C2C12 myotubes. This observation is in line with previous literature, showing that defects in muscle BCAA oxidation contribute to impaired lipid metabolism [33,34]. Interestingly, in addition to BCAA deprivation, high level of BCAAs also resulted in reduced lipid oxidation when compared to normal level of BCAAs. These results are supported by a previous study where both deprivation and excess supplementation of leucine reduced palmitate oxidation in C2C12 myotubes [35].

We observed that the deprivation of leucine or isoleucine but not valine alone reduced lipid oxidation. Lipid oxidation was also lower in the absence of isoleucine compared with the leucine or valine deprivation groups. In a previous study, upregulation of leucine catabolism together with β-oxidation was shown to protect mice from high-fat diet-induced obesity, supporting the role of leucine catabolism on lipid oxidation and metabolic health [36]. In addition, isoleucine supplementation seems to simultaneously activate liver and skeletal muscle free fatty acid uptake and oxidation [19]. The observed prominent effect of isoleucine on lipid oxidation in our study may partly be due to the fact that, unlike valine or leucine, isoleucine can feed the tricarboxylic acid (TCA) cycle through two separate pathways: via acetyl-CoA or succinyl-CoA [3], thus, perhaps, acting as a regulator of TCA activity. In the case of valine, the α-ketoacids, intermediate products resulting from BCAA catabolism, undergo oxidative decarboxylation in the reactions catalyzed by BCKD. BCKD is a rate-limiting enzyme complex in BCAA oxidation, which is activated by increased availability of leucine and isoleucine but not valine [37]. If BCAA and lipid oxidation are interconnected, the lack of BCKD activation by valine could partly explain the smaller role of valine on lipid oxidation.

Our results also revealed that BCAA deprivation reduced lipogenesis in myotubes. Deprivation of a single BCAA at a time reduced lipogenesis compared with normal BCAA level, yet the decrease was not as significant as when all BCAAs were absent. These findings support our previous hypothesis that BCAA deprivation would not only disrupt lipid oxidation but also lipogenesis [3]. Supporting our hypothesis, studies have shown that BCAAs fuel lipogenesis in adipocytes and adipose tissue [38,39]. Furthermore, removing BCAAs from culture media prevents the ATP synthase-mediated LD formation in murine myocytes [40]. According to our hypothesis, increased BCAA catabolism is essential for cytosolic oxaloacetate production that is metabolized to phosphoenolpyruvate for glyceroneogenesis. Increased glyceroneogenesis in turn leads to the storage of fatty acids in lipid droplets in myocytes. Deprivation of BCAAs seems to disrupt this cycle, leading to reduced lipogenesis. We observed decreased BCAAs and their breakdown products using NMR spectroscopy, suggesting indeed substantially decreased BCAA degradation when BCAAs are absent from the C2C12 culture medium.

### 3.2. EPS Treatment Decreased Lipid Oxidation but Not Lipogenesis in C2C12 Myotubes

In the present study, we further examined the effect of EPS on lipid oxidation and lipogenesis in murine C2C12 myotubes. Our results revealed that EPS decreased lipid oxidation, especially when combined with high BCAA supplementation. Accordingly, we have previously reported that EPS reduces lipid oxidation under normal BCAA conditions [41]. This result is probably due to the very glycolytic nature of C2C12 cells in ATP production, also during low-intensity EPS [41,42]. In this setup, we did not observe a difference in lipid oxidation between BCAA deprivation and normal BCAA groups, possibly due to small n used in the EPS experiments (*n* = 4) compared with the BCAA-deprivation experiments (*n* = 21–26).

Furthermore, the high BCAA supplementation combined with EPS-reduced lipid oxidation. In previous literature, some studies have shown a decrease in intramuscular lipid content during electrically stimulated muscle contractions in situ or in vivo [43,44,45], while others have not observed a decrease [46]. These inconsistencies may be due to the high variability associated with the measurement of intramuscular lipid content as well as differences in the stimulation protocol and duration of the stimulation [44]. In vitro, EPS has been reported to have controversial effects on fatty acid oxidation, which may be explained, for example, by the methodological differences, cell line used or fatty acid analyzed [47,48,49]. In a previous study utilizing isolated soleus muscles, tetanic contractions increased palmitate oxidation [50]. However, in our setup, we were only able to successfully measure lipid oxidation. This is one limitation of our study, as lipid oxidation does not necessarily represent the whole lipid oxidation capacity of the myotubes. Furthermore, C2C12 cells normally use carbohydrates as their primary energy source [41,42]. Hence, cultured murine C2C12 myotubes with treated EPS do not necessarily fully represent muscle metabolism during human exercise in vivo.

### 3.3. BCAA Deprivation Diminished the Number of Lipid Droplets in the EPS-Treated C2C12 Myotubes

EPS treatment combined with BCAA deprivation diminished the number of lipid droplets in murine C2C12 myotubes. This observation is in line with previous literature showing that LDs are an important source of energy during exercise and that acute exercise as well as electrically stimulated muscle contractions reduce LDs due to increased oxidation [43,44,45,51]. Yet, this result is inconsistent with the findings in the present study that both BCAA deprivation and EPS led to decreased lipid oxidation. However, it is important to note that lipid oxidation directly concerns fatty acids and only indirectly LDs, which are made primarily of triacylglycerol [45].

As previously studied, a decrease in the LD number may be a reflection of fission, not only from chemical hydrolysis but from mechanical stress as well [52,53]

Nevertheless, despite the decrease in the number of detectable lipid droplets, the overall lipid signal was not affected by our experiments. This may be explained by the fact that a large part of neutral lipids (i.e., triacylglycerols) diffuse through the cytosol in the form of suboptical (<~100 nm in diameter) LDs [54]. We thus hypothesize that, when combined with myotube contractions, BCAA-driven lipogenesis could be providing fatty acids for triacylglycerol esterification and respective LD replenishment. Conversely, BCAA deprivation and consequent reduced lipogenesis would lead to lower fatty acid availability, resulting in lower esterification rates and a reduced number of LDs. An overall reduced lipid oxidation explains the unchanged neutral lipid signal.

## 4. Materials and Methods

Murine C2C12 myoblasts (American Type Culture Collection, ATCC, Manassas, VA, USA) were maintained in high glucose-containing Dulbecco’s Modified Eagle growth medium (GM) (4.5 g/L, DMEM, #BE12-614F, Lonza, Basel, Switzerland) supplemented with 10% (*v*/*v*) fetal bovine serum (FBS, #10270, Gibco, Rockville, MD, USA), 100 U/mL penicillin, 100 µg/mL streptomycin (P/S, #15140, Gibco) and 2 mM L-Glutamine (#17-605E, Lonza). For the experiments, myoblasts were seeded on 12-well or 6-well plates (NunclonTM Delta; Thermo Fisher Scientific, Waltham, MA, USA). When the myoblasts reached 95–100% confluence, the cells were rinsed with phosphate-buffered saline (PBS, pH 7.4), and the GM was replaced by differentiation medium (DM) containing high glucose DMEM, 2% (*v*/*v*) horse serum (HS, 12449C, Sigma-Aldrich, St. Luis, MO, USA), 100 U/mL and 100 µg/mL P/S and 2 mM L-glutamine to promote differentiation into myotubes, unless stated otherwise. Fresh DM was changed every other day. The cells were screened negative for mycoplasma contaminations, following manufacturer’s instructions (MycoSPY Master Mix Test Kit, M020, Biontex, München, Germany). The experiments were conducted on days 5–6 post differentiation, and samples were collected immediately after indicated time points.

### 4.1. Treatments

The BCAA deprivation and supplementation experiments were carried out in high-glucose BCAA-free DM, i.e., containing no L-leucine, L-isoleucine and L-valine. More specifically, DM contained high glucose BCAA-free DMEM (4.5 g/L, BioConcept, 1-26S289-I, Allschwill, Switzerland), 2% (*v*/*v*) horse serum, 100 U/mL penicillin, 100 µg/mL streptomycin and 2 mM L-Glutamine. The experimental groups were as follows: normal BCAA cells (normal BCAA) were supplemented with 0.8 mmol/L of all BCAAs, relative to which BCAA deprivation (0 mmol/L, no BCAA), and high BCAA supplementation (2.8 mmol/L, high BCAA) groups contained indicated amounts of BCAAs. In deprivation of a single BCAA at the time, the media were deficient of either L-leucine, L-isoleucine or L-valine and were supplied with 0.8 mmol/L of other BCAAs. The experiments were repeated in duplicate or triplicate.

#### 4.1.1. Lipid Oxidation

Lipid oxidation experiments were carried out by measuring oleate oxidation as described previously [41]. Briefly, for measuring oleate oxidation, the murine C2C12 myotubes grown in normal BCAA conditions (0.8 mml/l) were first acclimatized to dissolved and albumin-complexed 0.1 mM oleic acid (#O3008, Sigma-Aldrich, St. Luis, MO, USA) and 1 mM L-carnitine (C0158, Sigma-Aldrich) in DM on the day 4 post differentiation. The following day, the myotubes were rinsed with PBS and incubated in the oxidation medium containing BCAA-free medium, 0.1 mM oleic acid, 2% (*v*/*v*) horse serum, 100 U/mL penicillin, 100 µg/mL streptomycin and 2 mM L-Glutamine, 1 mM L-carnitine and 1 µCi/mL [9,10-^3^H(N)] oleic acid (24 Ci/mmol, NET289005MC, PerkinElmer, Boston, MA, USA). The radiolabeled oleic acid was omitted from the negative controls. The lipid oxidation experiment was carried out for 2 h at 37 °C, as previously described [41]. Target BCAA concentration (no BCAA, normal BCAA or high BCAA) was applied during the 2 h lipid oxidation experiment to investigate the effect of BCAA deprivation or supplementation on lipid oxidation. After the lipid oxidation experiment, the myotubes were washed with PBS and also PBS was collected. The myotubes were harvested into PBS-0.1% PBS-Triton X-100 for enzyme activity and total protein content analyses. The media and PBS were run through Dowex OH resin ion-exchange columns (pH 7.1 × 8^−200^, Cat no. 217425, Sigma Aldrich). Deionized H_2_O was used to elute the ^3^H_2_O produced and secreted by the myotubes to the media. The radioactivity was analyzed as disintegration per minute (DPM), as previously described [41]. The radioactivity that had been incorporated in ^3^H_2_O was determined by scintillation counting in Optiphase HiSafe 3 scintillation cocktail (Cat no. 1200.437, PerkinElmer) with Tri-Carb 2910 TR Liquid Scintillation Analyzer (PerkinElmer) and expressed as DPM/per well. The lipid oxidation results were expressed relative to normal BCAA group.

#### 4.1.2. Skeletal-Muscle-Specific Exercise-like Electrical Pulse Stimulation (EPS) and Lipid Oxidation

The murine C2C12 myotubes on 6-well plates were acclimatized to 0.1 mM oleic acid and 1 mM L-carnitine in normal BCAA DM on the day 4 post differentiation. On the next day, the electrodes were placed directly onto the wells. The electrical stimulation (1 Hz, 2 ms, 12 V) was applied to the cells using a C-Pace pulse generator (C-Pace EM, IonOptix, Milton, MA, USA) for 24 h at 37 °C with the same protocol as described earlier [41], as it has been shown to mimic various exercise-like responses [41,48]. As described previously, EPS was paused after 22 h and lipid oxidation experiment was carried out for 2 h at 37 °C with EPS and target BCAA concentration (no BCAA, normal BCAA or high BCAA) to investigate the interactive effects of EPS and BCAA deprivation or supplementation on lipid oxidation. For the final 10 min of EPS, 10 min control myotubes were supplied with the radiolabeled medium. The 10 min controls were used as a baseline, and the control DPM was subtracted from the 2 h measurement DPMs. After the EPS, the oxidation media were collected. A 500 µL aliquot was taken from the medium, from which 20 µL input was placed on scintillation vials. The remaining 480 µL were run through Dowex OH resin ion-exchange columns and the ^3^H_2_O produced by the cells was eluted with deionized H_2_O. The results were calculated as described above. The myotubes were harvested immediately after the EPS into PBS-0.1% PBS-Triton X-100 for enzyme activity and total protein content analyses. The lipid oxidation results were expressed relative to normal BCAA group.

#### 4.1.3. Lipogenesis

Lipogenesis was measured by the uptake of ^3^H-acetate into the lipids described previously [55], with minor modifications. On the day 5 post differentiation, murine C2C12 myotubes were washed with PBS and changed to DM with target BCAA concentration (no BCAA, normal BCAA or high BCAA) to investigate the effect of BCAA deprivation or supplementation on lipogenesis. In addition, DM was supplied with 10 µM sodium acetate (Cat no. 32319, Sigma-Aldrich, USA) and 0.5 µCi ^3^H-acetate (Cat.no. NET003H005MC, PerkinElmer, USA) per well to stimulate lipogenesis. Myotubes were incubated for 16 h at 37 °C in lipogenesis medium. After incubation, cells were washed with PBS and scraped into 0.1 M HCl. An aliquot of the lysate was reserved for analysis of total protein content. The lipids were extracted with 2:1 chloroform/methanol (v:v) [55]. The samples were centrifuged for 10 min at 3000 x g in RT. The lower phases were transferred to scintillation vials. The radioactivity that had been incorporated in the cellular lipids was determined by scintillation counting, as described above. The results were expressed relative to normal BCAA group.

#### 4.1.4. Electrical Pulse Stimulation (EPS) and Lipogenesis

On the day 5 post differentiation, the murine C2C12 myotubes on 6-well plates were washed with PBS and fresh lipogenesis medium with target BCAA concentration (no BCAA, normal BCAA or high BCAA) as described above was added. Electrical stimulation (1 Hz, 2 ms, 12 V) was applied for 16 h. After the EPS, the cells were collected for lipid extraction. The scintillation counting was performed as described above. The lipogenesis results were expressed relative to normal BCAA group.

### 4.2. Histology and Image Analysis

Histology and image analysis were carried out for lipid oxidation experiments. For this purpose, the cells were cultured on 6-well plates containing coverslips. Each experimental group was measured from 18 coverslips. After the 24 h of EPS, the cells were fixed with 4% paraformaldehyde for 15 min, followed by 30 min blocking using 10% goat serum (GS) in PBS with 0.05% saponin (PBS-SAP). As a marker for differentiated murine C2C12 myotubes, MF-20 mouse monoclonal antibody (Developmental Studies Hybridoma Bank, University of Iowa, Iowa City, IA) was incubated for 1 h with a dilution 1:100 in 1% GS in PBS-SAP. The secondary antibody incubation with Alexa fluor 647 Donkey anti-Mouse IgG (H+L) (Thermo Fisher Scientific) for 1 h with a 1:150 dilution in 1% GS in PBS-SAP was followed by marking of the neutral lipids and nuclei for 30 min in PBS with 0.1 μg/mL of LD540 [56] and 0.5 μg/mL of DAPI (Thermo Fisher Scientific), respectively. The samples were mounted with Mowiol. The confocal images (voxel size = 0.1 × 0.1 × 1.3 µm) were made from three random 203.3 × 203.3 µm areas in each coverslip, using a Zeiss LSM700 microscope with a 63×/1.4 oil objective. Nuclei, myotubes and neutral lipid markers were excited with a 405, 488 and 555 laser line, respectively. The MF-20 signal was used to segment and analyze lipid droplets from differentiated myotubes only. Image analysis was performed in ImageJ 1.53c and Python 3.9.0. Cell culture, microscopy and image analysis were performed blindly from each other.

### 4.3. Nuclear Magnetic Resonance (NMR) Spectroscopy

The cell lysates and the experiment media were collected and prepared for the ^1^H-NMR analysis from lipid oxidation experiment. The method and data analysis have been explained in detail previously [41]. Briefly, media from three wells were mixed with cold methanol (600 μL of sample and 1.200 μL of methanol), and cells were scraped into 200 µL of 90% (v:v) 9:1 aqueous methanol/chloroform mixture. The resulting supernatants were lyophilized and then reconstituted. All the NMR spectra were collected using a Bruker AVANCE III HD NMR spectrometer, operating at 800 MHz ^1^H frequency (Bruker Corporation, MA, USA) equipped with a cryogenically cooled ^1^H, ^13^C, ^15^N triple-resonance probe head. The obtained data were analyzed using Chenomx software 8.6 (Chenomx, Edmonton, AB, Canada).

### 4.4. Total Protein Content and Enzyme Activity Measurements

Total protein content (Bicinchoninic Acid Protein Assay Kit, Pierce Biotechnology, Rockford, IL, USA) and citrate synthase (CS) activity (#CS0720, Sigma-Aldrich) were analyzed according to the manufacturers’ protocol with an automated Indiko analyzer (Thermo Fisher Scientific, Vantaa, Finland). To assess the cell viability, media lactate dehydrogenase (LDH) activity was measured from the EPS experiments using an LDH assay kit following the manufacturer’s instructions (#981906, Thermo Fisher Scientific, Waltham, Massachusetts, Canada) [57].

### 4.5. Statistical Analyses

The results in the figures are presented as interquartile ranges and medians with 95% confidence interval (CI) and in tables as mean and standard error of mean (SEM). The normal distribution of the variables was assessed using Shapiro–Wilks tests followed by Levene’s test for examining the equality of the variances. First, the extreme outliers were excluded from the analysis (>3× interquartile range). In the lipogenesis and lipid oxidation experiments, the data are expressed relative to control group (normal BCAA), since the treatments significantly affected the total protein content and CS activity of the samples (Supplementary Figures S1–S3). When the normality criteria were met, the differences between the groups were examined using one-way ANOVA followed by independent samples *t*-test. When the normality criteria were not fulfilled, the differences between the groups were examined using Kruskal–Wallis test followed by Mann–Whitney U-test. In the NMR results, groups differing from BCAA level and EPS treatment were compared using Mann–Whitney U-test. Data analyses were carried out using IBM SPSS Statistics. In all analyses, *p*-value < 0.05 was considered to indicate statistical significance.

## 5. Conclusions

The present study shows that deprivation of BCAAs reduces both lipid oxidation and lipogenesis in cultured murine C2C12 myotubes. These results partially support our previous hypothesis that, in skeletal muscle, adequate BCAA catabolism increases lipid oxidation and lipogenesis when compared to BCAA deprivation. However, it appears that a higher BCAA level is not able to further increase these processes; instead, it may even affect them contrariwise. Exercise mimicking EPS decreased lipid oxidation, especially when combined with high BCAA supplementation, whereas BCAA deprivation combined with EPS diminished the number of lipid droplets in myotubes. Our results highlight the role of an adequate level of BCAAs in a healthy lipid metabolism.

## Data Availability

Data is contained within the article or Supplementary Materials.

## Supplementary Materials

The following supporting information can be downloaded at: https://www.mdpi.com/article/10.3390/metabo12040328/s1, Table S1: Metabolites measured via ^1^H-NMR spectroscopy in the myotubes and the cell culture media (µM); Table S2: List of all identified metabolites in the myotubes and the cell culture media via ^1^H-NMR spectroscopy; Figure S1: The C2C12 myotube total protein content (a) and CS activity (b) in lipid oxidation experiment and total protein content in lipogenesis experiment (c) with different BCAA concentrations; Figure S2: The C2C12 myotube total protein content (a) and CS activity (b) in lipid oxidation experiment and total protein content (c) in lipogenesis experiments with BCAA deprivation; Figure S3: The C2C12 myotube total protein content (a,c) and CS activity (b,d) in lipid oxidation experiments with no and high BCAA and 24h EPS and protein content (e) in lipogenesis experiment with no BCAA and 16h EPS; Figure S4: LDH activity (U/l) in cell media with and without EPS and with different BCAA levels in lipid oxidation (a) and lipogenesis (b) experiments.
