# Supplementary material for: Branched-Chain Amino Acid Deprivation Decreases Lipid Oxidation and Lipogenesis in C2C12 Myotubes
Source: Metabolites. 2022 Apr 5;12(4):328. doi: 10.3390/metabo12040328 (PMC9031053; doi:10.3390/metabo12040328)
Supplement: Supplementary file 1 [file metabolites-12-00328-s001.zip › metabolites-1630704-supplementary.pdf]

Supplementary material

Supplementary Table S1. Metabolites measured via ^{1}H-NMR spectroscopy in the myotubes and the cell culture media (μM). Data is presents as mean (SEM).

	Metabolite (μM)	No BCAA control	No BCAA EPS	Normal BCAA control	Normal BCAA EPS	p (BCAA level)	p (EPS)
Myotubes (n=3/group)	Pooled BCAAs	6.6 (3.8)	14.0 (8.1)	213.9 (123.5)	275.8 (159.2)	**0.004**	0.749
	Isoleucine	2.0 (1.2)	4.4 (2.6)	75.7 (43.7)	97.4 (56.2)	**0.004**	0.749
	Leucine	2.3 (1.3)	4.7 (2.7)	66.0 (38.1)	85.4 (49.3)	**0.004**	0.749
	Valine	2.3 (1.3)	4.8 (2.8)	72.2 (41.7)	93.0 (53.7)	**0.004**	0.749
	Pooled BCAA degradation products	0.0 (0.0)	0.0 (0.0)	2.2 (1.3)	2.1 (1.2)	0.140	0.902
	Isobutyrate	0.0 (0.0)	0.0 (0.0)	0.3 (0.2)	0.3 (0.2)	0.138	1.000
	Isovalerate	0.0 (0.0)	0.0 (0.0)	0.9 (0.5)	0.7 (0.4)	0.140	0.902
	2-Methylbutyrate	0.0 (0.0)	0.0 (0.0)	1.0 (0.6)	1.1 (0.7)	0.140	0.902
Cell media (n=2-3/group)	Pooled BCAAs	23.4 (16.5)	36.9 (26.1)	1717.3 (1214.3)	1789.0 (1032.9)	**0.014**	0.806
	Isoleucine	8.2 (5.8)	12.7 (9.0)	629.1 (444.8)	646.0 (372.9)	**0.014**	0.806
	Leucine	6.8 (4.8)	10.9 (7.7)	475.5 (336.2)	509.5 (294.1)	**0.014**	0.806
	Valine	8.4 (6.0)	13.3 (9.4)	612.8 (433.3)	633.6 (365.8)	**0.014**	1.000
	Pooled BCAA degradation products	6.6 (4.6)	10.6 (7.5)	46.9 (33.2)	46.8 (27.0)	**0.014**	1.000
	Isobutyrate	0.7 (0.5)	0.7 (0.5)	3.6 (2.5)	2.1 (1.2)	0.121	1.000
	Isovalerate	1.2 (0.8)	1.1 (0.7)	10.2 (7.2)	6.8 (4.0)	0.121	1.000
	2-Ketoisovalerate	0.8 (0.6)	1.5 (1.1)	3.5 (2.5)	5.1 (2.9)	0.348	0.184
	2-Methylbutyrate	1.3 (0.9)	1.3 (0.9)	10.3 (7.3)	5.5 (3.2)	0.121	1.000
	3-Methyl-2-oxovalerate	1.5 (1.1)	3.3 (2.3)	9.3 (6.6)	13.5 (7.8)	0.355	0.127
	2-Oxoisocaproate	1.1 (0.8)	2.7 (1.9)	10.0 (7.1)	13.8 (8.0)	0.355	0.275

Supplementary Table S2. List of all identified metabolites in the myotubes and the cell culture media via ^{1}H-NMR spectroscopy.

	Identified (yes=x)	
Metabolite	Myotubes	Media
Acetate	x	x
ADP	x	
Alanine	x	x
β-Alanine	x	
Arginine	x	x
Aspartate	x	
Carnitine	x	x
Choline		x
Citrate	x	x
Creatine	x	
Creatine phosphate	x	
Cystine		x
Formate	x	x
Fumarate	x	
Glucose	x	x
Glutamate	x	
Glutamine	x	x
Glycerol	x	x
Glycine	x	x

GTP	x	
Guanidoacetate	x	
Histidine		x
Isobutyrate	x	x
Isoleucine	x	x
Isovalerate	x	x
2-Ketoisovalerate		x
Lactate	x	x
Leucine	x	x
Lysine	x	x
Malate	x	
Methionine	x	x
2-Methylbutyrate	x	x
3-Methyl-2-oxovalerate		x
Myo-Inositol	x	x
NAD+	x	
Niacinamide		x
O-Acetylcarnitine	x	
O-Phosphocholine	x	
2-Oxoisocaproate		x
Pantothenate	x	x
Phenylalanine	x	x
Propionate	x	
Pyridoxine		x
Pyroglutamate		x
Pyruvate	x	x
Serine	x	x
sn-Glycero-3-phosphocholine	x	
Succinate	x	
Taurine	x	
Threonine	x	x
Tryptophan		x
Tyrosine	x	x
UDP-N-Acetylglucosamine	x	
UDP-glucose	x	
Valine	x	x

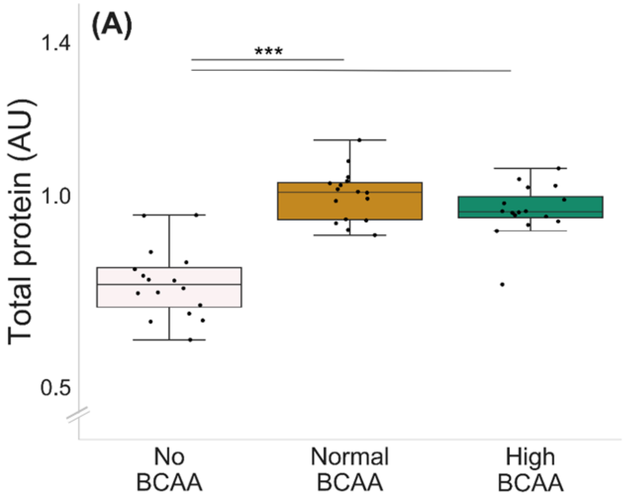

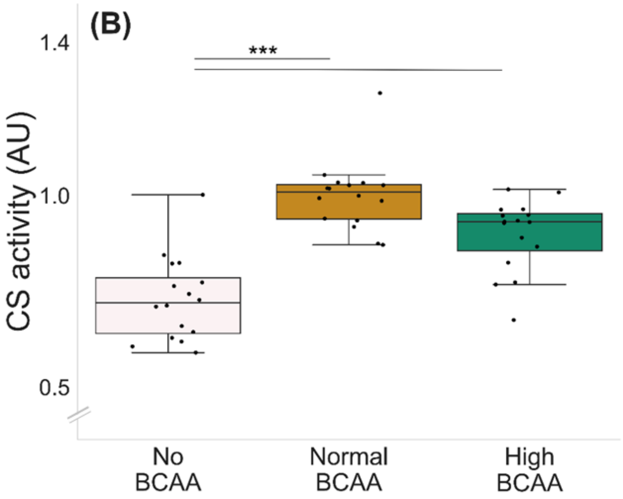

(a) (b)

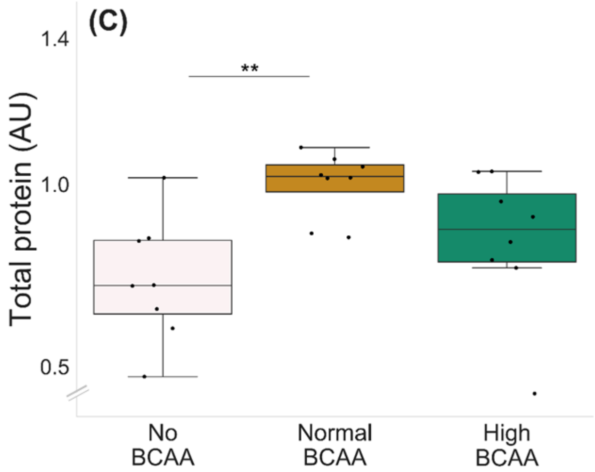

(c)

Supplementary Figure S1. The C2C12 myotube total protein content **(a)** and CS activity **(b)** in lipid oxidation experiment and total protein content in lipogenesis experiment **(c)** with different BCAA concentrations. **(a)** Compared with No BCAA, both Normal (0.8 mmol/l) and High (2.8 mmol/l) of BCAA groups had higher total protein content. n=16/group. **(b)** No BCAA group had lower CS activity compared with Normal and High BCAA groups. n=16/group. **(c)** Compared with No BCAA, Normal BCAA (0.8 mmol/l) group had higher protein content. n=8/group. Boxes in the boxplot figures depict interquartile ranges and medians, whiskers represent the 95% confidence interval, **p≤0.010, ***p≤0.001. n=16/group. BCAA=branched-chain amino acid, CS=citrate synthase.

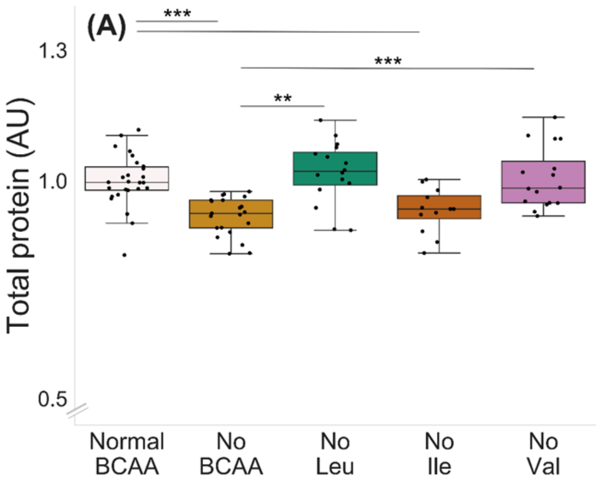

(a)

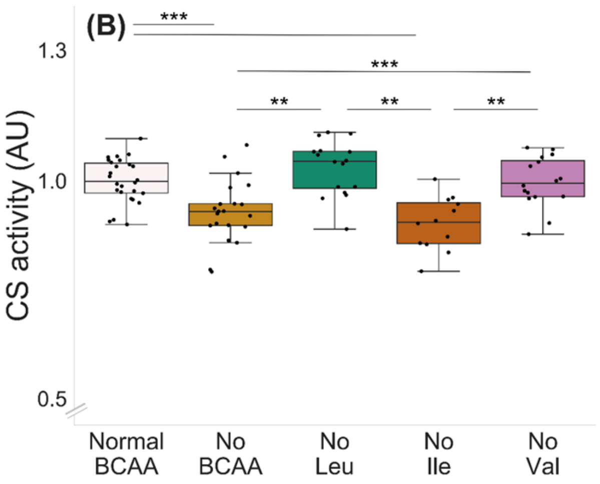

(b)

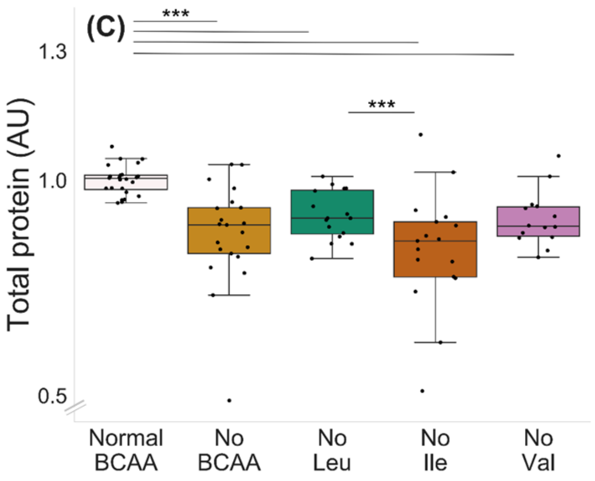

(c)

Supplementary Figure S2. The C2C12 myotube total protein content **(a)** and CS activity **(b)** in lipid oxidation experiment and total protein content **(c)** in lipogenesis experiments with BCAA deprivation. **(a)** BCAA deprivation reduced protein content of the samples in No BCAA and isoleucine deprivation groups. No BCAA group had also lower protein content compared with leucine and valine deprivation groups. Normal BCAA n=26, No BCAA n=21, No leu n=16, No ile n=12, No val n=16. **(b)** Deprivation of all BCAAs or isoleucine alone reduced CS activity compared with Normal BCAA (0.8 mmol/l) group. No BCAA group had lower CS activity compared with leucine and valine deprivation groups and isoleucine deprivation lead to lower CS activity compared with leucine or valine deprivation. Normal BCAA n=26, No BCAA n=21, No leu n=16, No ile n=12, No val n=16. **(c)** BCAA deprivation reduced protein content of the samples. Isoleucine deprivation led to lower protein content compared with leucine deprivation. Normal BCAA n=24, No BCAA n=23, No leu n=14, No ile n=17, No val n=14. Boxes in the boxplot figures depict interquartile ranges and medians, whiskers represent the 95% confidence interval, $^{*}p<0.050$, $^{**}p\leq0.010$, $^{***}p\leq0.001$. BCAA=branched-chain amino acid, Leu=leucine, Ile=isoleucine, Val=valine, CS=citrate synthase.

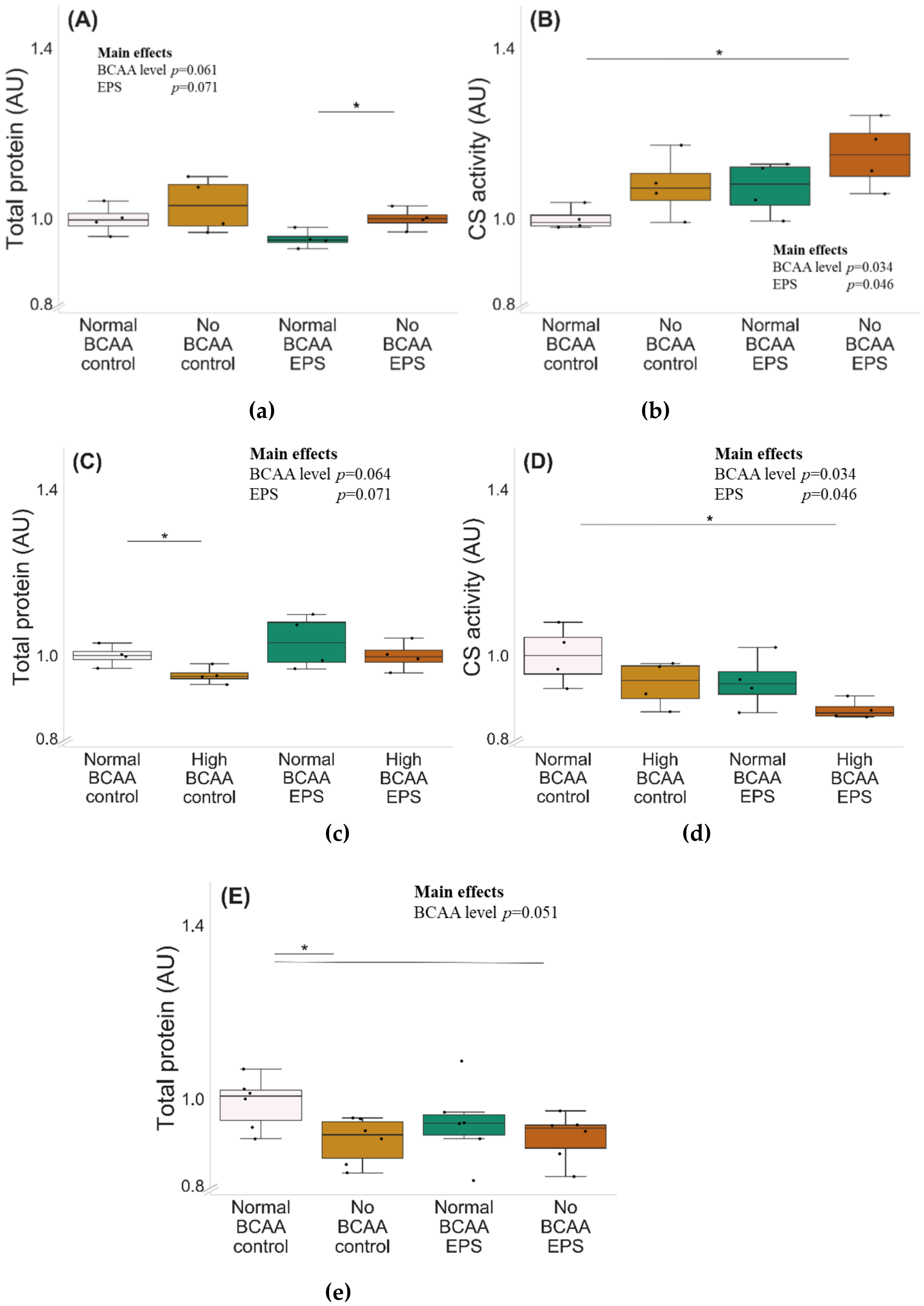

Supplementary Figure S3. The C2C12 myotube total protein content **(a,c)** and CS activity **(b,d)** in lipid oxidation experiments with No and High BCAA and 24h EPS and protein content **(e)** in lipogenesis experiment with no BCAA and 16h EPS. **(a)** BCAA deprivation reduced protein content of No BCAA EPS group compared with Normal BCAA (0.8 mmol/l) EPS group. **(b)** CS activity was higher in No BCAA EPS group compared with Normal BCAA group. **(c)** High BCAA (2.8 mmol/l) reduced protein content compared with Normal BCAA (0.8 mmol/l) control group. **(d)** CS activity was lower in high BCAA EPS group compared with Normal BCAA control group. **(e)** Compared with the corresponding control groups, BCAA deprivation led to reduced lipogenesis. n=4/group. Boxes in the boxplot figures depict interquartile ranges and medians, whiskers represent the 95% confidence interval, *p<0.050, **p≤0.010, ***p≤0.001. n=4/group. BCAA=branched-chain amino acid, EPS=skeletal muscle-specific exercise-like electrical pulse stimulation, CS=citrate synthase.

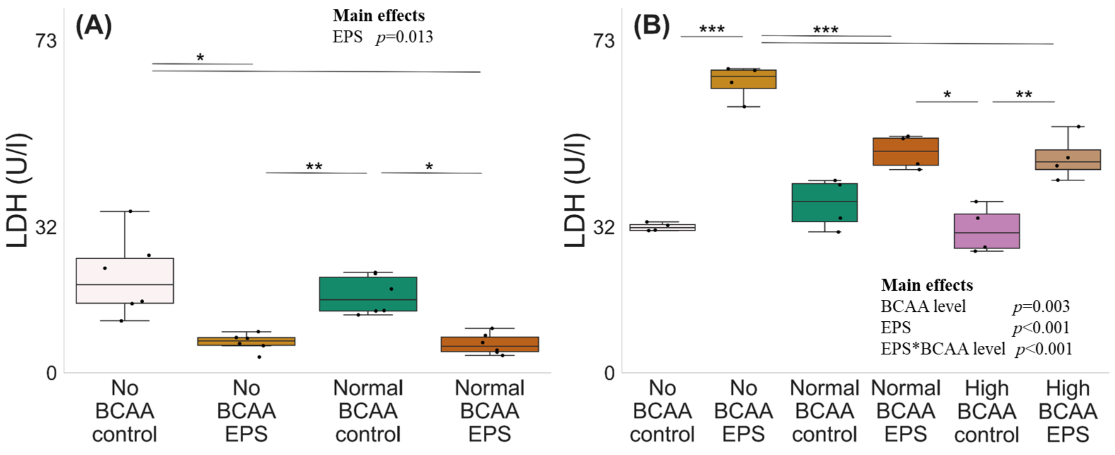

(a) **(b)**

Supplementary Figure S4. LDH activity (U/l) in cell media with and without EPS and with different BCAA levels in lipid oxidation **(a)** and lipogenesis **(b)** experiments. **(a)** EPS decreased LDH activity in cell media in lipid oxidation experiment (main effect of EPS p=0.013) n=6/group. **(b)** EPS increased LDH activity in all of the studied BCAA concentrations in lipogenesis experiment (main effect of EPS<0.001). BCAA concentration had an effect on LDH activity (p=0.003) and there was an interaction of EPS and BCAA level (p<0.001). Boxes in the boxplot figures depict interquartile ranges and medians, whiskers represent the 95% confidence interval, *p<0.050, **p≤0.010, ***p≤0.001. n=4/group. BCAA=branched-chain amino acid, EPS=skeletal muscle-specific exercise-like electrical pulse stimulation. LDH= lactate dehydrogenase.
